# Allomyrinasin, an Edible Insect-Derived Peptide, Ameliorates High-Fat Diet-Induced Hepatic Oxidative Stress and Metabolic Dysfunction

**DOI:** 10.3390/antiox15060755

**Published:** 2026-06-15

**Authors:** Kyong Kim, Chae-Heon Lee, Chae-Eun Kim, Eun-Young Park, Jae-Sam Hwang, Yoon Sin Oh

**Affiliations:** 1 Department of Food and Nutrition, Eulji University, Seongnam 13135, Republic of Korea; kim_kyong@daum.net (K.K.); cogjs4112@naver.com (C.-H.L.); kce0224@naver.com (C.-E.K.); 2 College of Pharmacy and Natural Medicine Research Institute, Mokpo National University, Jeoannam, Muan-gun 58554, Republic of Korea; parkey@mokpo.ac.kr; 3 Future Food Research Institute, Hanmi Nutrition, Paju 10808, Republic of Korea; hwangjsrda63@daum.net

**Keywords:** *Allomyrina dichotoma* larva, allomyrinasin, high-fat diet, hepatic lipogenesis, oxidative stress, liver metabolism

## Abstract

Allomyrinasin is an antimicrobial peptide derived from the larvae of the edible insect Allomyrina dichotoma and has been reported to exert anti-inflammatory activity, although its role in metabolic regulation remains unclear. This study aimed to investigate the metabolic and hepatoprotective effects of allomyrinasin in a high-fat diet (HFD)–induced obese mouse model. Male C57BL/6J mice were fed an HFD for 6 weeks to induce body weight gain and hyperglycemia, followed by 10 weeks of oral administration of allomyrinasin (0.1 mg/kg/day) under continued HFD conditions, with metformin used as a positive control. Metabolic parameters related to glucose homeostasis, insulin sensitivity, lipid metabolism, hepatic injury, oxidative stress, inflammation, and fibrotic responses were evaluated. Allomyrinasin significantly attenuated HFD-associated body weight gain and improved glucose tolerance and insulin sensitivity. These effects were accompanied by favorable modulation of serum lipid profiles and suppression of hepatic lipogenic signaling, including reduced expression of key regulators of de novo lipogenesis. In parallel, allomyrinasin mitigated hepatic inflammatory, fibrotic, and oxidative stress-related alterations, as reflected by improvements in biochemical markers and molecular analyses. Collectively, these findings indicate that allomyrinasin contributes to the improvement of metabolic regulation and hepatic homeostasis in HFD-fed mice. Our results support allomyrinasin as a promising food-applicable bioactive peptide and potential functional ingredient for the prevention or management of obesity-related metabolic disorders.

## 1. Introduction

The growing burden of metabolic diseases, including obesity, type 2 diabetes mellitus (T2DM), hypertension, and dyslipidemia, and their strong association with cardiovascular and hepatic dysfunction, represents a major global health problem [1,2,3]. In the search for effective therapeutics and prevention to alleviate these metabolic diseases, natural products have long served as a rich source of bioactive compounds, those derived from plants.

In recent years, insect-derived biomolecules have gained increasing attention as a promising new frontier often described as a “blue ocean” owing to their remarkable structural diversity, unique chemical properties, and multifunctional biological activities [4,5]. In particular, phytophagous species are capable of sequestering, modifying, and reassembling plant-derived secondary metabolites such as flavonoids, terpenoids, alkaloids, and phenolics, leading to the formation of structurally novel and functionally distinct molecules [6,7]. This metabolic conversion endows insects with tremendous potential as a source of innovative bioactive compounds, many of which may remain undiscovered within conventional natural product research.

Recent research has demonstrated that insects produce a diverse range of antimicrobial peptides (AMPs) and secondary metabolites, which function not only as potent antibiotics but also as agents with anti-inflammatory, antioxidant, and immunomodulatory activities [8,9]. Building on these biological properties, recent research has revealed that certain insect-derived compounds can ameliorate metabolic dysfunctions, including insulin resistance, dyslipidemia, and hepatic steatosis, through the regulation of inflammatory signaling and oxidative stress pathways [10,11,12,13]. These findings suggest that insect-derived ‘metabolic syndrome targeting agent’ may confer metabolic benefits beyond host defense, highlighting their potential as novel candidates for metabolic disease intervention.

One of the edible insects, the *Allomyrina dichotoma* larvae, has long been used in East Asian folk medicine, and accumulating studies indicate that larval extracts exert beneficial metabolic effects. Our previous study demonstrated that *A. dichotoma* larva extract (ADLE) ameliorates hepatic insulin resistance in high-fat diet (HFD)-induced diabetic mice, through activation of AMPK and suppression of lipogenic gene expression [13]. These findings strongly suggest that larval-derived compounds possess potent antidiabetic and lipid-regulatory activities. However, the specific bioactive molecules responsible for these effects remained undefined.

Allomyrinasin, a cationic antimicrobial peptide, was first identified and characterized by its amino acid sequence (AAVTRRILCWFA-NH_2_), using de novo transcriptome sequencing of *A. dichotoma* larvae [14]. It was reported that it has broad-spectrum antimicrobial activity against both Gram-positive and Gram-negative bacteria, and potent anti-inflammatory properties in lipopolysaccharides (LPS) stimulated macrophages [14]. Subsequent studies further confirmed the dual antimicrobial and anti-inflammatory activities of allomyrinasin, thereby extending its pharmacological potential beyond host defense. Given the central role of chronic low-grade inflammation in the pathogenesis of insulin resistance and dyslipidemia [15,16,17], bioactive peptides such as allomyrinasin, may offer therapeutic benefits in metabolic disorders. Nevertheless, its antidiabetic and lipid-modulatory effects have not been systematically investigated in vivo.

Therefore, in this study, we examined the metabolic effects of allomyrinasin in a high-fat diet-induced diabetic mouse model, with metformin employed as a positive control. By assessing the effects of relatively low doses of allomyrinasin on glucose homeostasis, insulin sensitivity, and lipid profiles, we aimed to provide new insights into the potential of insect-derived antimicrobial peptides as dual-function therapeutics for metabolic diseases.

## 2. Materials and Methods

### 2.1. Allomyrinasin Synthesis

The synthetic peptide allomyrinasin (AAVTRRILCWFA-NH_2_) was custom-synthesized by HLB Peptide Co., Ltd. (Gwangju, Republic of Korea) using a solid-phase peptide synthesis method. The peptide was obtained as a white amorphous powder. Purity (95.1%) was verified by analytical reverse-phase HPLC using a Dikma Inspire C_18_ column (5 µm) with a 5–65% B buffer gradient over 30 min (A buffer: 0.05% TFA/H_2_O; B buffer: 0.05% TFA/acetonitrile), flow rate of 1 mL/min, detection at 230 nm, and column temperature of 35 °C. Purity was calculated based on peak area normalization. Molecular mass was confirmed by MALDI-TOF mass spectrometry (AXIMA Assurance, AXIMA Assurance, Shimadzu, Kyoto, Japan), showing a major peak at *m*/*z* 1405.7 Da, consistent with the theoretical mass (1405.8 Da). All analytical data, including (A) HPLC chromatograms and (B) MALDI-TOF mass spectra, were provided by the manufacturer (Supplementary Figure S1). The peptide was dissolved in sterile distilled water at a concentration of 1.0 mg/mL immediately prior to use.

### 2.2. Animal Model and Treatment Protocol

All animal procedures were approved by the Eulji University Institutional Animal Care and Use Committee (EUIACUC-24-07, 30 July 2024). Six-week-old male mice (corresponding to a human adolescent age of approximately 12–15 years) [18] were purchased from Central Lab. Animal Inc. (Seoul, Republic of Korea) and acclimated for one week under controlled environmental conditions (22 ± 2 °C, 12 h light/dark cycle) with free access to food and water. The experimental period concluded when the mice reached 23 weeks of age, which corresponds to a young human adult (approximately 20–26 years of age). This study is a randomized controlled animal experiment with a genotype-stratified, body weight-matched, completely randomized design, where the experimental unit is a cage of animals. Body weight was set as an important monitored parameter throughout the study, given its close association with the metabolic phenotype of high-fat-fed mice. After acclimatization, the animals were randomly divided into two groups: a normal diet (ND) group (*n* = 8) and 60% high-fat diet (HFD, D12492, Research Diets, Inc., New Brunswick, NJ, USA) group (*n* = 24). After the 6-week induction period, the HFD-fed mice were subdivided into three treatment groups (*n* = 8 per group) according to their body weight and fasting blood glucose levels, using a randomized block design to minimize baseline variation among groups. The groups were as follows: (1) the normal diet (ND) group (*n* = 8), which was maintained on a standard chow diet as model of the healthy control; (2) the high-fat diet (HFD) group (*n* = 8), which continued on the 60% high-fat diet without treatment as the disease control; (3) the HFD + allomyrinasin group (*n* = 8), which received allomyrinasin (0.1 mg/kg/day, orally) to assess its metabolic effects; (4) the HFD + metformin group (*n* = 8), which received metformin (Cayman Chemical, Cayman Chemical, Ann Arbor, MI, USA) (100 mg/kg/day, orally) as a positive control. Allomyrinasin and metformin were dissolved in sterile distilled water and administered once daily by oral gavage using a gavage needle, while the ND and HFD control groups received the same volume of sterile distilled water as the vehicle. The dose of allomyrinasin was chosen as a conservative exploratory low dose for this first long-term oral in vivo study, considering previous findings on oral bioactive peptides and the 10-week repeated administration schedule [19,20].

After grouping, an independent samples *t*-test was used to verify that there was no statistically significant difference in baseline mean body weight between the control and treatment groups within each stratum (*p* > 0.05), confirming successful body weight matching. To avoid additional environmental confounding, the cage positions of all mice were rotated every two weeks to ensure uniform distribution of each group on the animal rack. The order of body weight measurement was also determined according to a random number table to eliminate systematic errors caused by operation order.

A total of 32 mice were used in the final analysis. The sample size of the total enrolled animals was determined based on the well-validated settings of the same HF-fed mouse model in peer-reviewed published studies [18]. This setting fully covers the needs of multi-index detection at different experimental endpoints, ensures sufficient statistical test power for all analyses, and strictly complies with the Reduction requirement of the 3R principles for laboratory animal welfare.

For food intake and food efficiency ratio (FER), the cage was defined as the experimental unit (*n* = 3) cages per group, containing 3, 3, and 2 mice, respectively, calculated by dividing the total cage consumption by the number of co-housed mice. For all other physiological, biochemical, and histological parameters, the individual mouse was considered the independent experimental unit (*n* = 8) per group.

This study adopted a single-blind design for allocation concealment and outcome assessment. The random sequence, grouping table, and drug coding were generated by a researcher who did not participate in animal feeding, experimental operation, or data analysis. The vehicle and treated peptides were prepared as reagents with identical appearance, volume, and administration frequency with blind codes. The operators performing animal feeding, administration, and body weight measurement were blinded to the grouping information throughout the experiment. All body weight data recording, histopathological section reading, biochemical index detection, and data statistical analysis were completed by researchers who were completely blinded to the grouping information. The grouping information was unblinded only after all experimental data collection and statistical analysis were completed.

All treatments were administered once daily for 10 weeks while maintaining the same dietary conditions. Body weight and food intake were recorded weekly, and fasting blood glucose levels were measured every two weeks after a 4 h fast to monitor metabolic changes during the experimental period. General health and behavior were also observed throughout the study. The criteria for excluding animals and data points were established a priori before the commencement of the study. Animal exclusion criteria: Animals were excluded from the analysis if they died during the experiment, exhibited significant weight loss (more than 20%), or showed signs of infection.

### 2.3. Food Efficiency Ratio (FER)

Food efficiency ratio (FER, %) was calculated as the total body weight gain divided by the cumulative food intake during the 10-week treatment period, multiplied by 100. Because food intake was monitored on a cage basis, the cage was treated as the experimental unit (*n* = 3) cages per group, housing 3, 3, and 2 mice, respectively. Accordingly, cage-level FER was determined by dividing the average body weight gain per mouse by the average cumulative food intake per mouse within each respective cage.

### 2.4. Glucose and Insulin Tolerance Tests

A glucose tolerance test (GTT) was performed at week 9 after a 12 h fast. Mice were injected intraperitoneally with glucose (Sigma Aldrich, St. Louis, MO, USA, 2 g/kg body weight), and blood glucose was measured at 0, 30, 60, and 120 min. An insulin tolerance test (ITT) was conducted at week 10 following a 4 h fast. Insulin (Sigma Aldrich, 1.5 IU/kg body weight) was injected intraperitoneally, and blood glucose levels were determined at the same time points by Caresens II plus^+^ glucometer (i-SENS, Incheon, Republic of Korea). The area under the curve (AUC) was calculated for both tests.

### 2.5. Biochemical and Metabolic Analyses

Serum glucose, albumin, blood urea nitrogen (BUN), triglycerides (TG), total cholesterol (TC), high-density lipoprotein (HDL), aspartate aminotransferase (AST), and alanine aminotransferase (ALT) levels were determined using commercial assay kits (Asan Pharmaceutical, Seoul, Republic of Korea) according to the manufacturer’s instructions. Low-density lipoprotein (LDL) concentrations were calculated using the Friedewald formula as follows: LDL (mg/dL) = TC − HDL − (TG/5). Serum insulin and whole blood HbA1c levels were quantified using ELISA-based assay kits (Crystal Chem, Elk Grove Village, IL, USA). The homeostatic model assessment for insulin resistance (HOMA-IR) was calculated using the following formula [21]: HOMA-IR = [fasting glucose (mg/dL) × fasting insulin (ng/mL) × 28.7]/405, where fasting insulin values measured in ng/mL were converted to µU/mL using the conversion factor of 1 ng/mL ≈ 28.7 µU/mL. All biochemical measurements were performed in duplicate, and the results were expressed as mean ± SD. All measurements were conducted with a multimode plate reader (VICTOR Nivo, PerkinElmer, Waltham, MA, USA).

### 2.6. Hepatic Lipid Accumulation and Lipid Peroxidation

Liver tissues (approximately 25 mg) were homogenized in 10 volumes of ice-cold phosphate-buffered saline (PBS, pH 7.4) with 0.01% BHT. The homogenates were centrifuged at 13,000× *g* for 10 min at 4 °C, and the supernatants were collected for analysis. To measure hepatic lipid accumulation, triglyceride (TG) content was quantified using a commercial biochemical assay kit (Asan Pharmaceutical, Seoul, Republic of Korea) and expressed as mg TG per g of wet liver tissue. Hepatic lipid peroxidation was quantified using a lipid peroxidation (MDA) assay kit (Abcam, Cambridge, UK, ab233471) according to the manufacturer’s protocol. The MDA concentration in each sample was calculated using a standard curve and normalized to total protein content (expressed as nmole MDA per mg).

### 2.7. Hematoxylin and Eosin Staining

Liver tissues were fixed in 10% neutral-buffered formalin (pH 6.8–7.2; Simport, Beloeil, QC, Canada), embedded in paraffin, and sectioned at 10 µm thickness. The sections were stained with hematoxylin and eosin (H&E) for histopathological evaluation. H&E staining was used to assess hepatic steatosis, inflammatory cell infiltration, and hepatocellular ballooning. Histological changes were evaluated using the NAFLD Activity Score (NAS) system, which is defined as the sum of steatosis (0–3), lobular inflammation (0–3), and hepatocellular ballooning (0–2). For quantitative analysis, each image was subdivided into four non-overlapping regions, and histological features were independently assessed in each region to increase sampling resolution. All stained sections were imaged using an Olympus BX61 microscope equipped with an Olympus DP70 digital camera (Olympus Co., Tokyo, Japan). Image brightness and contrast were uniformly adjusted for visualization without altering the original information.

### 2.8. Quantitative Real-Time PCR Analysis

Total RNA was extracted from liver tissues using a TRIzol-based method (Invitrogen, Grand Island, NY, USA). Complementary DNA (cDNA) was synthesized using a reverse transcription kit (Takara Bio Inc., Takara Bio Inc., Kusatsu, Shiga, Japan). Quantitative real-time polymerase chain reaction (*q*RT-PCR) was performed using gene-specific primers and SYBR Premix Ex Taq II (ROX plus; Takara Bio Inc., Shiga, Japan) according to the manufacturer’s instructions. Lipid metabolism-related genes included sterol regulatory element-binding protein 1*c* (*SREBP-1c*), fatty acid synthase (*FAS*), and acetyl-CoA carboxylase (*ACC*). Inflammatory markers analyzed were tumor necrosis factor-α (*TNF-α*), interleukin-6 (*IL-6*), and monocyte chemoattractant protein-1 (*MCP-1*). For fibrosis and tissue injury, the expression of alpha-smooth muscle actin (*α-SMA*), collagen, fibronectin, and transforming growth factor-β1 (*TGF-β1*) was evaluated. Antioxidant and stress-related genes included cytochrome P450 2E1 (*CYP2E1*) and glutathione peroxidase 4 (*GPx4*). Primer sequences used for the analysis are listed in Table 1, and relative mRNA expression levels were normalized to β-actin and calculated using the 2^ΔΔCt^ method, with the results expressed as fold changes relative to the ND group.

### 2.9. Western Blot Analysis

Total protein was extracted from liver tissues using Cellytic lysis buffer (Thermo Fisher Scientific, Waltham, MA, USA) supplemented with protease and phosphatase inhibitor cocktails (Sigma-Aldrich, St. Louis, MO, USA). The lysates were incubated on ice for 30 min and then centrifuged at 12,000× *g* for 15 min at 4 °C to remove debris. The supernatants were collected, and protein concentrations were determined using the Bradford assay kit (Thermo Fisher Scientific, Waltham, MA, USA).

Equal amounts of protein (20 µg) were separated by SDS–polyacrylamide gel electrophoresis (SDS-PAGE) and transferred onto nitrocellulose membranes (MilliporeSigma, Burlington, MA, USA). Membranes were blocked with 3% non-fat dry milk in Tris-buffered saline containing 0.1% Tween 20 (TBST) for 1 h at room temperature and then incubated overnight at 4 °C with primary antibodies against target proteins related to lipid metabolism (SREBP-1, FAS, and ACC), inflammation (COX2), fibrosis (fibronectin, COL1A1, and TGF-β1), and oxidative stress (4-HNE). After washing with TBST, the membranes were incubated with appropriate horseradish peroxidase (HRP)-conjugated secondary antibodies for 2 h at room temperature. The immunoreactive bands were detected using an enhanced chemiluminescence (ECL) detection system (Bio-Rad Laboratories, Hercules, CA, USA), visualized with an ATTO WSE-6200 LuminoGraph II imaging system (ATTO Corporation, Tokyo Japan), and quantified using CS Analyzer 4 software (ATTO Corporation, Tokyo, Japan).

### 2.10. Statistical Analysis

All data are expressed as the mean and standard deviations (SD). Statistical significance was analyzed using SPSS 20.0 software (IBM SPSS ver. 20.0.0 for Windows; IBM Co., Armonk, NY, USA). Significant differences among the groups were analyzed using the LSD comparisons test. Statistical significance was set at *p <* 0.05.

## 3. Results

### 3.1. Allomyrinasin Modulates Body Weight Gain and Food Efficiency Ratio During HFD Feeding

**Figure 1:**
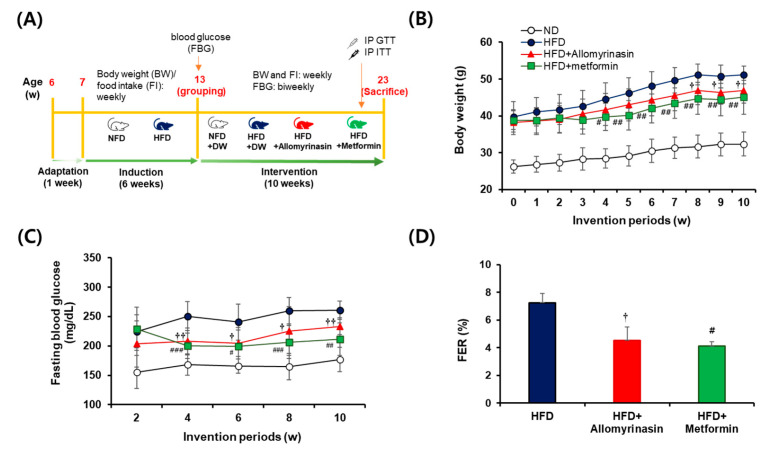
Experimental design and metabolic parameters in HFD-induced mice treated with allomyrinasin. (**A**) Schematic overview of the experimental procedure. (**B**) Body weight was monitored weekly for 10 weeks in each group. (**C**) Fasting blood glucose levels were measured bi-weekly for 10 weeks. (**D**) Food efficiency ratio (FER, %) was calculated during the 10-week treatment period and compared only among HFD-fed groups maintained on the same diet. Data are presented as mean ± SD (*n* = 8 mice per group for B and C; *n* = 3 cages per group for D). ^†^ *p* < 0.05 and ^††^ *p* < 0.01 (HFD + allomyrinasin vs. HFD); ^#^ *p* < 0.05, ^##^ *p* < 0.01, and ^###^ *p* < 0.01 (HFD + metformin vs. HFD). Significant differences were observed in body weight (**B**) and fasting blood glucose (**C**) at all time points between the ND and HFD groups (*p* < 0.001), and the significance level was omitted for the readability of the graph. ND: normal diet, HFD: high-fat diet; FER: food efficiency ratio.

As shown in Figure 1A, after 6 weeks of 60% HFD feeding, mice exhibited approximately 1.5-fold higher body weight compared with ND controls (*p* < 0.001) (Figure 1B). During treatment administration of allomyrinasin (0.1 mg/kg/day) or metformin (100 mg/kg/day; positive control) effectively suppressed further HFD-induced weight gain. The anti-obesity effect of allomyrinasin became significant from week 8 onward (*p* < 0.05), whereas metformin showed an earlier response at week 4. At the end of the study, body weight in the allomyrinasin group was reduced by 8.4% relative to the HFD group (*p* < 0.05), comparable to that observed in the metformin-treated group (Figure 1B). In parallel, allomyrinasin significantly lowered fasting blood glucose levels from week 4 onward, showing glucose-lowering efficacy comparable to metformin by the end of the experiment (*p* < 0.01 vs. HFD) (Figure 1C). Additionally, allomyrinasin significantly reduced FER compared with the HFD group (*p* < 0.05), reaching a level comparable to that of the metformin-treated group (Figure 1D). Together with the reductions in body weight and fasting blood glucose, these results suggest that allomyrinasin attenuated HFD-induced metabolic dysfunction.

### 3.2. Allomyrinasin Ameliorates Glucose and Insulin Intolerance in HFD-Fed Mice

**Figure 2:**
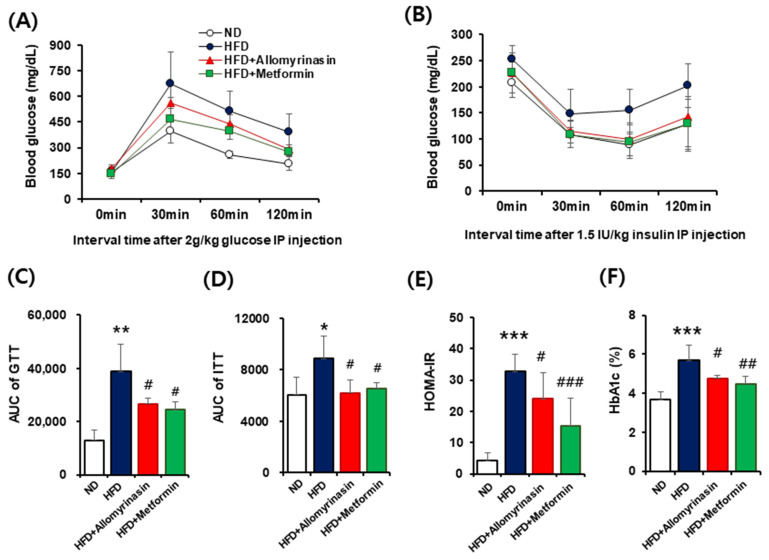
Effects of allomyrinasin on glucose homeostasis in HFD-fed mice. (**A**) Glucose tolerance test (GTT) was performed after 12 h of fasting. Blood glucose levels were measured at 0, 30, 60, and 120 min following glucose administration (2 g/kg, i.p.). (**B**) Insulin tolerance test (ITT) was conducted after 4 h of fasting. Blood glucose levels were recorded at 0, 30, 60, and 120 min following insulin injection (1.5 IU/kg, i.p.). (**C**) Area under the curve (AUC) was calculated from the blood glucose response curves. (**D**) AUC was calculated to evaluate insulin sensitivity. (**E**) Homeostatic model assessment of insulin resistance (HOMA-IR). (**F**) Glycated hemoglobin (HbA1c) levels. Data are shown as mean ± SD (n = 8). * *p* < 0.05, ** *p* < 0.01 and *** *p* < 0.001 vs. ND group; ^#^ *p* < 0.05, ^##^ *p* < 0.01, and ^###^ *p* < 0.001 vs. HFD group.

To further assess glucose homeostasis after allomyrinasin treatment, GTT and insulin ITTs were performed at weeks 9 and 10, respectively. In the GTT (Figure 2A), HFD-fed mice showed markedly impaired glucose clearance compared with the ND group, as indicated by significantly higher blood glucose levels and increased AUC (*p* < 0.001, Figure 2B). Allomyrinasin treatment significantly improved glucose tolerance, resulting in a reduced AUC compared with the HFD group (*p* < 0.05), comparable to that of the metformin group. In the ITT, HFD-fed mice exhibited a blunted hypoglycemic response to insulin, indicating reduced insulin sensitivity. Both allomyrinasin and metformin improved insulin responsiveness, as reflected by lower glucose levels and reduced AUC values compared with the HFD group (*p* < 0.05, Figure 2C,D). Consistently, HOMA-IR values were significantly elevated in HFD-fed mice (*p* < 0.001) but markedly decreased following allomyrinasin treatment (*p* < 0.05), similar to the metformin-treated group (Figure 2E). Moreover, HbA1c levels were significantly higher in the HFD group (*p* < 0.001) but were reduced after allomyrinasin administration (*p* < 0.05), consistent with improvements in HOMA-IR (Figure 2F). Together, these findings demonstrate that allomyrinasin alleviates HFD-induced glucose intolerance and insulin resistance, and enhances long-term glycemic control to an extent comparable with metformin.

### 3.3. Allomyrinasin Restores Lipid Homeostasis in HFD-Fed Mice

**Figure 3:**
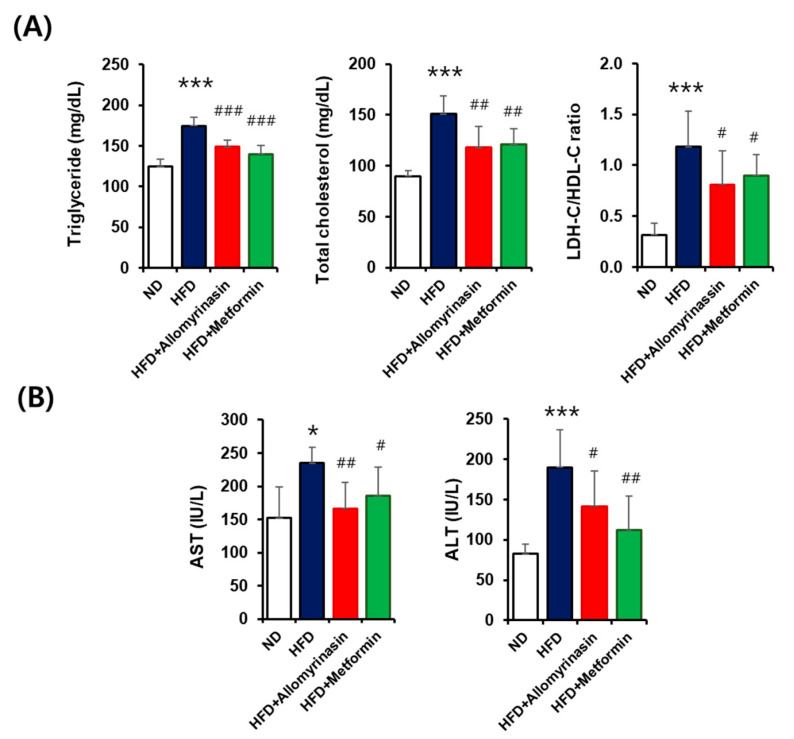
Effects of allomyrinasin on serum lipid profiles and hepatic biomarkers in HFD-fed mice. (**A**) Serum triglyceride (TG), total cholesterol (TC), and the ratio of low-density lipoprotein (LDL-C) to high-density lipoprotein (HDL-C). (**B**) Aspartate aminotransferase (AST), and alanine aminotransferase (ALT) were analyzed in normal diet (ND), high-fat diet (HFD), HFD + allomyrinasin, and HFD + metformin. Data are expressed as mean ± SD. * *p* < 0.05, *** *p* < 0.001 vs. ND group; ^#^ *p* < 0.05, ^##^ *p* < 0.01, and ^###^ *p* < 0.001 vs. HFD group.

To determine whether the metabolic benefits of allomyrinasin extend to lipid metabolism, serum lipid profiles were evaluated (Figure 3A). HFD-fed mice displayed markedly elevated levels of TC, TG, and LDL-c/HDL-c ratio, compared with the ND group (*p* < 0.001). Allomyrinasin treatment significantly normalized these parameters, lowering TG (*p* < 0.001), TC (*p* < 0.01), and LDL-c/HDL-c ratio (*p* < 0.05) levels relative to the HFD group. The magnitude of these changes was comparable to that observed in the metformin-treated group, indicating similar lipid-modulating efficacy. To further examine hepatoprotective effects, serum levels of AST and ALT, indicators of hepatic injury, were measured. HFD-fed mice showed significantly elevated AST and ALT levels compared with ND controls (*p* < 0.001, Figure 3B), reflecting diet-induced hepatic stress. Allomyrinasin treatment markedly reduced AST (*p* < 0.01) and ALT (*p* < 0.05) levels, comparable to reductions observed with metformin. These findings suggest that allomyrinasin protects against HFD-induced hepatic injury while restoring lipid homeostasis.

### 3.4. Allomyrinasin Attenuates HFD-Induced Hepatic Lipid Accumulation and Oxidative Damage

**Figure 4:**
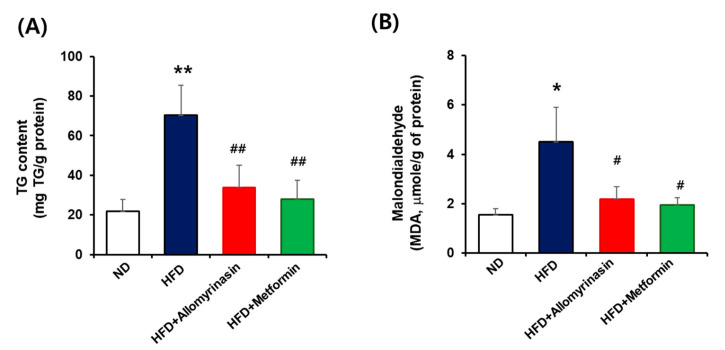
Effects of allomyrinasin on hepatic triglyceride (TG) and malondialdehyde (MDA) levels in liver tissue of HFD-fed mice. (**A**) Hepatic triglyceride (TG) levels. (**B**) Hepatic MDA levels, an indicator of lipid peroxidation. Data are expressed as mean ± SD (*n* = 8). * *p* < 0.05 and ** *p* < 0.01 vs. ND group. ^#^ *p* < 0.05 and ^##^ *p* < 0.01 vs. HFD group.

Following the evaluation of serum AST and ALT, hepatic TG and MDA levels were analyzed to further assess lipid accumulation and oxidative stress in liver tissue (Figure 4). Hepatic TG levels were markedly elevated in HFD-fed mice, showing approximately a 3.2-fold increase compared with the ND group (*p* < 0.01), indicative of severe hepatic lipid deposition. Allomyrinasin administration significantly reduced hepatic TG levels by around 75% relative to the HFD group, comparable to the reduction observed with metformin treatment (Figure 4A). Similarly, hepatic MDA levels, a marker of lipid peroxidation, were increased by about 2.9-fold in the HFD group compared with ND controls (*p* < 0.05), reflecting enhanced oxidative stress. Allomyrinasin treatment markedly decreased MDA levels by approximately 78% (*p* < 0.05) relative to HFD-fed mice, and a comparable reduction (about 86%) was observed in the metformin-treated group (Figure 4B). Collectively, these findings suggest that allomyrinasin effectively mitigates hepatic lipid accumulation and oxidative stress induced by HFD, supporting its potent hepatoprotective and antioxidant properties.

### 3.5. Allomyrinasin Ameliorates HFD-Induced Hepatic Steatosis

**Figure 5:**
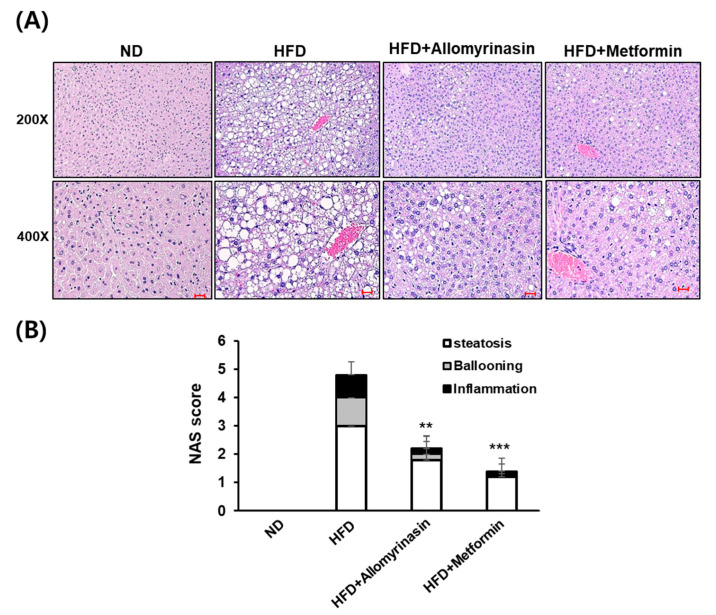
Histological assessment of liver tissues in HFD-fed mice. Liver sections were stained with hematoxylin and eosin (H&E) to evaluate hepatic morphology. (**A**) Representative hematoxylin and eosin (H&E)-stained liver sections from ND, HFD, HFD + allomyrinasin, and HFD + metformin groups. (**B**) Quantification of the NAFLD Activity Score (NAS) for each group. Data are presented as mean ± SD (n = 6). Scale bars = 400 μm. Data are expressed as mean ± SD. ** *p* < 0.01, *** *p* < 0.001 vs. HFD group.

As shown in Figure 5A, H&E staining revealed prominent macrovesicular steatosis in the livers of HFD-fed mice, characterized by large cytoplasmic lipid droplets displacing hepatocyte nuclei toward the periphery. In contrast, allomyrinasin treatment visibly reduced hepatic lipid droplet accumulation and partially restored hepatocellular architecture, with histological features comparable to those observed in the metformin-treated group. Consistent with these morphological findings, the total NAFLD Activity Score (NAS) was markedly elevated in the HFD group compared with the ND group . Both allomyrinasin (*p* < 0.01) and metformin (*p* < 0.001) treatments significantly reduced the composite NAS relative to the HFD group.

### 3.6. Allomyrinasin Modulates Hepatic Dysfunction Markers Under HFD Conditions

**Figure 6:**
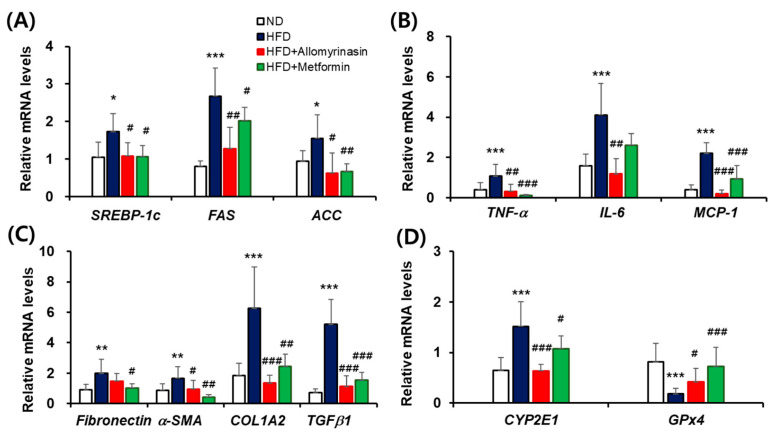
Effects of allomyrinasin on hepatic genes associated with metabolic dysfunction in HFD-fed mice. (**A**) Lipogenesis-related mRNA expression (SREBP-1c, FAS, ACC). (**B**) Inflammatory mediator mRNA expression (MCP-1, TNF-α, IL-6). (**C**) Fibrosis-related mRNA expression (fibronectin, α-SMA, COL1A2, TGF-β1). (**D**) Oxidative stress–related mRNA expression (CYP2E1, GPx4). Hepatic mRNA levels were quantified by quantitative real-time PCR in mice fed a normal diet (ND), high-fat diet (HFD), HFD supplemented with allomyrinasin, or metformin. Gene expression levels were normalized to an internal control gene (β-actin) and expressed relative to the ND group. Data are expressed as mean ± SD (*n* = 8). * *p* < 0.05, ** *p* < 0.01, and *** *p* < 0.001 vs. ND group. ^#^ *p* < 0.05, ^##^ *p* < 0.01 and ^###^ *p* < 0.001 vs. HFD group.

To investigate the effect of allomyrinasin on hepatic gene expression associated with metabolic dysfunction, we examined the transcriptional levels of key regulators related to lipogenesis, inflammation, fibrosis and oxidative stress. As shown in Figure 6A, the mRNA expression of lipogenic regulators including SREBP-1c, FAS, and ACC, was significantly upregulated in HFD group, increasing by 1.6-fold (*p* < 0.05), 3.3-fold (*p* < 0.001), and 1.7-fold (*p* < 0.05), respectively, compared with the ND group (Figure 6A). However, treatment with allomyrinasin markedly suppressed these HFD-induced increases to levels comparable to those observed in the metformin-treated group, indicating an effective inhibition of hepatic lipid biosynthesis.

**Figure 7:**
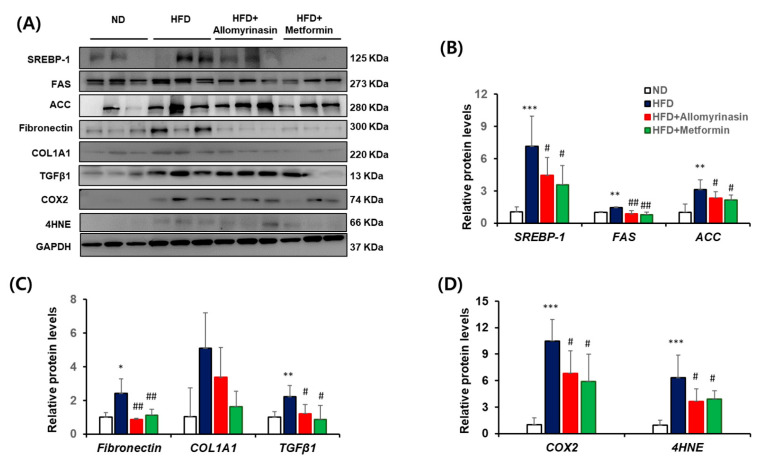
Effects of allomyrinasin on hepatic proteins associated with metabolic dysfunction in HFD-fed mice. (**A**) Representative Western blot images of hepatic proteins. (**B**) Densitometric analysis of lipogenesis-related proteins. (**C**) Densitometric analysis of fibrosis-related proteins. (**D**) Densitometric analysis of inflammation- and oxidative stress-related proteins. Protein expression of hepatic markers was analyzed by Western blotting in mice fed a normal diet (ND), high-fat diet (HFD), HFD supplemented with allomyrinasin, or metformin. Protein levels were normalized to GAPDH and expressed relative to the ND group. Data are expressed as mean ± SD (*n* = 3). ^#^ *p* < 0.05 and ^##^ *p* < 0.01 vs. HFD * *p* < 0.05, ** *p* < 0.01 and *** *p* < 0.001 vs. ND.

Furthermore, HFD feeding significantly increased the expression of inflammatory cytokines TNF-α, IL-6 and MCP-1, which are known to exacerbate metabolic dysfunction by promoting adipogenic and fibrogenic signals [22,23]. Their transcript levels were elevated by 2.7-fold, 2.6-fold, and 5.7-fold, respectively (Figure 6B). Allomyrinasin treatment markedly attenuated this inflammatory response, reducing the expression of all these mediators toward baseline levels. Consistent with increased inflammation, the expression of fibrosis-related genes such as fibronectin, α-SMA, COL1A2, and TGF-β1 was significantly upregulated in the HFD group compared with the ND group, showing increases of 2.2-fold (*p* < 0.01), 1.9-fold (*p* < 0.01), 3.4-fold (*p* < 0.001), and 7.3-fold (*p* < 0.001), respectively. Allomyrinasin treatment effectively attenuated the expression of these fibrotic markers (Figure 6C). As shown in Figure 6D, HFD also induced oxidative stress-related gene expression. The pro-oxidant enzyme CYP2E1, a major generator of ROS in fatty liver, was increased by 2.4-fold (*p* < 0.001) in HFD mice, whereas the antioxidant enzyme GPx4, essential for preventing lipid peroxidation and ferroptosis, was markedly reduced by 4.5-fold (*p* < 0.001). Allomyrinasin treatment normalized these alterations by reducing CYP2E1 expression and restoring GPx4 expression, demonstrating a strong hepatoprotective effect against ROS-induced injury. At the protein level, a similar expression pattern was observed. HFD feeding significantly increased protein levels of SREBP-1, FAS and ACC, compared with the ND group. Allomyrinasin treatment significantly suppressed these HFD-induced elevations, comparable to the effects of metformin (Figure 7A,B), confirming its inhibitory role in hepatic de novo lipogenesis. To determine whether the transcriptional regulation of fibrosis-related genes was reflected at the protein level, fibrotic proteins were analyzed (Figure 7C). Although collagen protein levels did not differ statistically among groups, fibronectin protein expression was increased by 2.4-fold (*p* < 0.01), and TGF-β1 by 2.2-fold (*p* < 0.01), in HFD group compared with ND. Allomyrinasin treatment significantly reduced fibronectin (*p* < 0.01) and TGF-β1 (*p* < 0.05) protein expression, restoring them to levels comparable to the ND group. Inflammatory responses were further evaluated by COX-2 protein expression, a downstream effector and established marker of metabolic inflammation (Figure 7D). COX-2 levels were markedly elevated by more than 10-fold (*p* < 0.001) in the HFD group compared with ND. Allomyrinasin significantly decreased COX-2 expression by 39% (*p* < 0.05) relative to the HFD, whereas metformin reduced it by 49% (*p* < 0.05). In addition, hepatic 4-HNE accumulation, a marker of lipid peroxidation, was markedly elevated in the HFD group (*p* < 0.001 vs. ND), but significantly reduced by allomyrinasin treatment (*p* < 0.05 vs. HFD), confirming its antioxidant efficacy. Metformin also decreased 4-HNE levels, consistent with its known antioxidant role.

## 4. Discussion

NAFLD has emerged as a central hepatic manifestation of diet-driven metabolic syndrome, tightly coupled to insulin resistance and systemic lipid imbalance. Chronic exposure to an HFD or hypercaloric intake accelerates hepatic steatosis and metabolic disorders, thereby recapitulating key pathological features observed in humans [3,24].

In our previous study, an aqueous ethanolic extract of *Allomyrina dichotoma* larvae was evaluated in vitro and in vivo, providing convergent evidence for hepatoprotective and antidiabetic activity under HFD-driven metabolic stress [13]. In the present study, we advance this line of work by shifting from a heterogeneous extract to allomyrinasin, an antimicrobial peptide (AMP) isolated from *A. dichotoma* larvae, as a defined bioactive component.

Accordingly, we focused on in vivo whole-body metabolic markers to verify whether insect-derived antimicrobial peptide, allomyrinasin is sufficient to reproduce the previously observed whole-body metabolic improvement effects of crude extracts in a high-fat diet-induced metabolic disorder model. Consistently, allomyrinasin improved glucose homeostasis, lipid metabolism, and hepatic integrity under HFD conditions, supporting its contribution, at least in part, to the protective effects of *A. dichotoma* larvae components. Allomyrinasin attenuated HFD-induced body weight gain and reduced feed efficiency, suggesting diminished energy storage or increased energy expenditure rather than a primary effect on caloric intake. Notably, these changes occurred during the early phase of high-fat diet exposure, a period critical for the initiation of adiposity expansion and insulin resistance [25,26]. The concurrent improvement in fasting blood glucose levels indicates that allomyrinasin may mitigate early impairments in glucose handling, thereby delaying the progression toward metabolic dysfunction.

Allomyrinasin improved glucose tolerance and insulin sensitivity, as reflected by reduced HOMA-IR and HbA1c levels. Because chronic hyperglycemia and insulin resistance are key drivers of metabolic deterioration and subsequent organ damage [27]. These findings suggest that allomyrinasin exerts metabolically protective effects that extend beyond acute glycemic control and may contribute to long-term metabolic homeostasis. Furthermore, allomyrinasin effectively restored the imbalanced lipid profile induced by HFD. Elevated serum triglycerides, total cholesterol, and LDL-c/HDL-c ratio are well known to be major causes of ectopic lipid accumulation and lipotoxicity [28]. The normalization of serum lipid parameters observed in the allomyrinasin-treated group is thought to be due to a decrease in lipid influx into the liver, thereby alleviating hepatic lipid overload and metabolic stress. Indeed, biochemical analyses further revealed that allomyrinasin significantly reduced hepatic triglyceride accumulation and lipid peroxidation, as evidenced by decreased malondialdehyde level.

Histological evaluation further confirmed the hepatoprotective effects of allomyrinasin. Livers from HFD-fed mice exhibited pronounced macrovesicular steatosis and architectural disruption, whereas allomyrinasin treatment markedly reduced lipid droplet accumulation and partially preserved hepatocellular organization. These structural improvements were accompanied by significant reductions in serum AST and ALT levels, indicating improved liver function and reduced hepatocellular injury. At the molecular level, allomyrinasin modulated the expression of genes involved in multiple pathogenic axes of metabolic liver disease. Downregulation of key lipogenic regulators, including SREBP-1, FAS, and ACC, is consistent with reduced de novo lipogenesis, a major contributor to hepatic fat accumulation in insulin-resistant states [29]. Concurrently, inhibition of proinflammatory mediators and fibrotic markers suggests that allomyrinasin attenuates the hepatic inflammatory and fibrotic responses that drive disease progression [30,31].

In particular, TGF-β1, produced by leukocytes and hepatic stromal cells, has been reported as a central profibrotic cytokine that drives liver fibrosis by promoting extracellular matrix deposition (e.g., collagen and fibronectin) and inhibiting its degradation. Several studies have consistently reported increased expression of TGF-β1 in models of HFD-induced metabolic liver disease, suggesting that it serves as a key profibrotic mediator in the initiation and progression of HFD-associated liver fibrosis [32,33,34]. The marked reduction of TGF-β1 mRNA and protein expression by allomyrinasin suggests effective disruption of a central fibrogenic signaling pathway, highlighting its therapeutic potential for mitigating HFD-induced hepatic fibrosis.

Oxidative stress plays a pivotal role in the progression from simple steatosis to steatohepatitis and fibrosis by amplifying inflammatory and fibrogenic signaling pathways [35]. CYP2E1 is a major hepatic source of reactive oxygen species (ROS) and is often upregulated under lipid overload and insulin resistance, thereby amplifying oxidative stress and lipid peroxidation in fatty liver disease [36,37]. In contrast, GPx4 detoxifies lipid hydroperoxides and protects cellular membranes from peroxidative injury, serving as a key regulator of lipid peroxidation–associated cellular damage [38,39]. Consistent with prior evidence linking CYP2E1-driven ROS production and impaired GPx4 defense to toxic lipid peroxidation products [39,40,41], allomyrinasin decreased hepatic *Cyp2e1* mRNA and increased *Gpx4* mRNA under HFD conditions, supporting improved hepatic redox homeostasis. This interpretation is further supported by biochemical evidence showing reduced hepatic triglyceride (TG) accumulation together with decreases in malondialdehyde (MDA) and 4-HNE levels, collectively indicating attenuation of lipid-driven oxidative injury in the steatotic liver [40,42].

Lipid overload-induced oxidative injury can trigger inflammatory and fibrogenic signaling in steatotic livers [43,44]. The observed suppression of inflammatory mediators by allomyrinasin, along with its regulation of oxidative stress, further supports its hepatoprotective role. Key pro-inflammatory cytokines (TNF-α, IL-6) and the chemokine MCP-1 drive hepatic inflammation by recruiting immune cells and amplifying cytokine cascade, thereby worsening metabolic dysregulation in fatty liver disease [15,30,45]. In this study, HFD increased cytokine expression and COX-2 levels, whereas allomyrinasin treatment attenuated both, indicating coordinated inhibition of cytokine-driven inflammatory signaling. As an inducible enzyme central to prostaglandin synthesis, COX-2 mediates chronic hepatic inflammation; thus, its suppression by allomyrinasin may help prevent the progression from steatosis to steatohepatitis by limiting persistent inflammatory and fibrogenic responses [31,46].

Several limitations should be noted. First, a comprehensive dose–response assessment of allomyrinasin was not performed, and potential toxicity at higher doses remains unexamined. In this study, a relatively low dose was administered over 10 weeks, chosen to ensure sustained efficacy under a safety-focused and physiologically relevant regimen for chronic metabolic disorders. It should be noted that our findings regarding the alterations in genes and proteins associated with lipogenesis, inflammation, fibrosis, and oxidative stress primarily demonstrate strong associations rather than direct mechanistic causality. Further functional studies, such as in vitro hepatocyte experiments and pathway inhibition assays, are warranted to validate the precise direct mechanisms. Moreover, future work should define the dose–response relationship and therapeutic window through higher-dose evaluation and systematic toxicity studies and further elucidate the underlying mechanisms using targeted cellular and pathway analyses. Lastly, although allomyrinasin possesses notable antimicrobial properties, its potential collateral impact on the gut microbiota remains to be fully elucidated. Further comprehensive microbiome profile analyses are warranted to clarify whether allomyrinasin influences the gut microbial ecosystem and its associated metabolic pathways.

Despite these limitations, the present findings suggest that allomyrinasin improves HFD-induced metabolic dysfunction in association with restoration of hepatic metabolic function. Notably, normalization of hepatic lipid handling and redox/inflammatory status was accompanied by improved glucose tolerance and insulin responsiveness, supporting the liver as a central mediator linking hepatoprotection to systemic glycemic control. Taken together, these findings suggest that allomyrinasin’s metabolic and hepatoprotective effects may be mediated by restoration of hepatic redox balance and attenuation of lipid peroxidation-associated injury, supporting its potential as a functional candidate for high-fat diet-induced metabolic disorders.

## 5. Conclusions

In conclusion, allomyrinasin, a larval antimicrobial peptide from *Allomyrina dichotoma*, restores hepatic metabolic homeostasis and improves systemic glucose regulation in high-fat diet–induced metabolic dysfunction. By modulating hepatic lipid metabolism, inflammation, fibrosis, and oxidative stress, allomyrinasin exerts metabolic and hepatoprotective effects comparable to metformin, without overt toxicity. These results position allomyrinasin as a promising bioactive candidate for the management of diet-associated metabolic disorders.

## Figures and Tables

**Table 1:** Primer sequence.

Target Gene	Accession No.	Forward (5′⟶3′)	Reverse (5′⟶3′)
*SREBP-1*	NM_011480	cttctggagacatcgcaaac	ggtagacaacagccgcatc
*FAS*	NM_007988	cttgggtgctgactacaacc	gccctcccgtacactcactc
*ACC*	NM_133360	aggaagatggcgtccgctctg	ggtgagatgtgctgggtcat
*COL1A2*	NM_007743	cttgccccattcatttgtct	ccgtgcttctcagaacatca
*TGFβ1*	NM_011577	ctcccgtggcttctagtgc	gccttagtttggacaggatctg
*α-SMA*	NM_007392	cccagattatgtttgagacc	cagagtccagcacaatacca
*Fibronectin*	NM_010233	tggaggagaaccaggagag	ggtgttgtaaggtggaatgg
*TNFα*	NM_013693	ccaacggcatggatctcaaagaca	agatagcaaatcggctgacggtgt
*IL-6*	NM_031168	taccacttcacaagtcggaggc	ctgcaagtgcatcatcgttgttc
*MCP-1*	NM_011333	gcagttaacgccccactca	ccagcctactcattgggatca
*CYP2E1*	NM_021282	aggctgtcaaggaggtgctact	aaaacctccgcacgtccttcca
*GPx4*	NM_008162	cctctgctgcaagagcctccc	cttatccaggcagaccatgtgc
*β-actin*	NM_007393	tgttaccaactgggaagaca	ggggtgttgaaggtctcaaa

## Data Availability

The datasets and materials used and/or analyzed during the current study are available from the corresponding author upon reasonable request.

## Supplementary Materials

The following supporting information can be downloaded at: https://www.mdpi.com/article/10.3390/antiox15060755/s1, Figure S1. High-performance liquid chromatography (HPLC) and mass spectrometric analysis of synthetic allomyrinasin.

## Abbreviations

The following abbreviations are used in this manuscript:

ACC	Acetyl-CoA carboxylase
AMP	Antimicrobial peptide
CYP2E1	Cytochrome P450 2E1
FAS	Fatty acid synthase
FER	Food efficiency ratio
GPx4	Glutathione peroxidase 4
GTT	Glucose tolerance test
HFD	High-fat diet
HOMA-IR	Homeostatic model assessment of insulin resistance
IL-6	Interleukin-6
ITT	Insulin tolerance test
MCP-1	Monocyte chemoattractant protein-1
MDA	Malondialdehyde
NAFLD	Non-alcoholic fatty liver disease
NAS	NAFLD activity score
ROS	Reactive oxygen species
HbA1c	Glycated hemoglobin
SREBP-1c	Sterol regulatory element-binding protein-1c
TGF-β1	Transforming growth factor-beta 1
TNF-α	Tumor necrosis factor-alpha
