# Supplementary material for: Allomyrinasin, an Edible Insect-Derived Peptide, Ameliorates High-Fat Diet-Induced Hepatic Oxidative Stress and Metabolic Dysfunction
Source: Antioxidants (Basel). 2026 Jun 15;15(6):755. doi: 10.3390/antiox15060755 (PMC13296046; doi:10.3390/antiox15060755)
Supplement: Supplementary file 1 [file antioxidants-15-00755-s001.zip › antioxidants-4330924-supplementary.pdf]

Supplementary Figure S1.

(A)

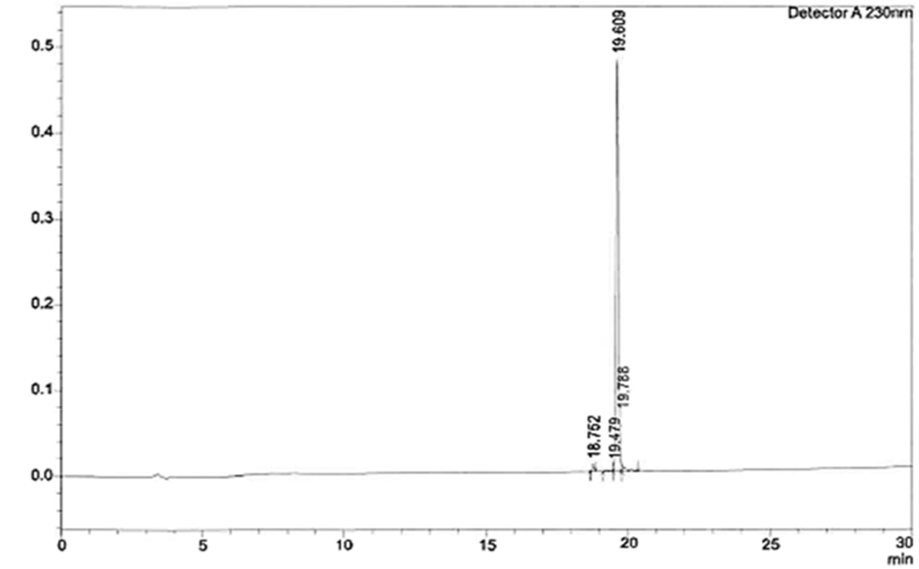


**<Peak Table>**

Detector A 230nm

| Peak# | Ret. Time | Area | Area% |
|---|---|---|---|
| 1 | 18.752 | 43029 | 1.414 |
| 2 | 19.479 | 20048 | 0.659 |
| 3 | 19.609 | 2893371 | 95.115 |
| 4 | 19.788 | 85523 | 2.811 |
| Total | | 3041971 | 100.000 |

(B)

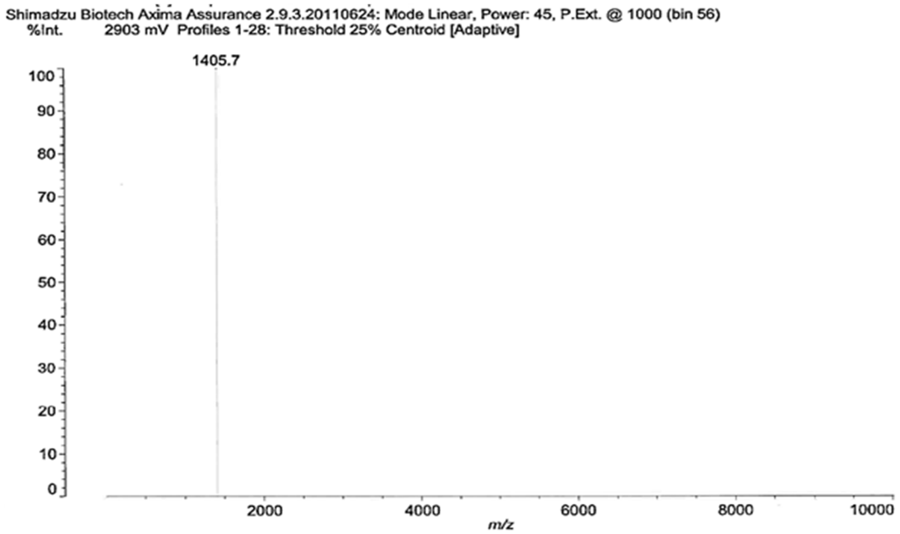


Figure S1. HPLC and mass spectrometric analysis of allomyrinasin. (A) HPLC chromatogram monitored at 230 nm. (B) Mass spectrum of the sample showing a major peak at m/z 1405.7.
